# Popliteal Artery Entrapment Syndrome Presenting with Acute Limb Ischaemia: A Case Report

**DOI:** 10.1155/2010/281925

**Published:** 2010-03-22

**Authors:** Ramawad Soobrah, Adam Nawaz, Tahir Hussain

**Affiliations:** ^1^Department of General Surgery, Northwick Park Hospital, Watford Road, Harrow HA1 3UW, UK; ^2^Faculty of Medicine, Imperial College London, South Kensington Campus, London SW7 2AZ, UK

## Abstract

Popliteal artery entrapment syndrome (PAES) is a relatively rare condition that occurs in young patients as a result of anomalous anatomic relationships between the popliteal artery and the surrounding musculotendinous structures. Patients usually lack atherogenic risk factors and most commonly present with intermittent claudication in the early stages. In the later stages of undiagnosed PAES, acute ischaemia can occur as a result of complete arterial occlusion or embolism. Hence, early diagnosis and surgical release of the entrapment is crucial for good operative outcome and to prevent limb loss.

## 1. Introduction

The anatomical basis of popliteal entrapment was first described by Stuart in 1879 [1] and the term “popliteal artery entrapment syndrome” was introduced by Love and Whelan in 1965 [2]. The condition is more common in men with a male-to-female ratio of 15 : 1 and can sometimes be bilateral [3]. Its true incidence is unknown but varies between 0.2%–3.5% [4]. PAES is mostly found in young sportsmen or soldiers with well-developed muscles and has even been described in military personnel who operate armored vehicles [5]. Typical patients are young and, therefore, lack the risk factors that would predispose them to atherosclerosis. The lack of such risk factors is the main reason for diagnostic delay [6]. The following case report outlines an unusual presentation of unilateral popliteal artery entrapment syndrome and delves into the importance of early diagnosis.

## 2. Case Report

A 24-year-old fit-and-healthy ex-army officer presented with a two-day history of a cold, numb, and painful left lower leg; he had been doing vigorous exercises two days prior to admission. Over the past few months, the patient had experienced similar worsening leg pain after running long distances. Apart from smoking (5 pack years), the patient had no other significant cardiovascular risk factors.

Physical examination revealed a regular heart rate of 60 beats per minute and a blood pressure of 120/80 mm Hg. There was no palpable abdominal aortic aneurysm. Examination of the right leg was unremarkable. On the left side, there were normal palpable femoral and popliteal pulses, but no palpable distal pulses. The left foot also appeared paler; however, motor and sensory functions were intact.

**Figure 1:**
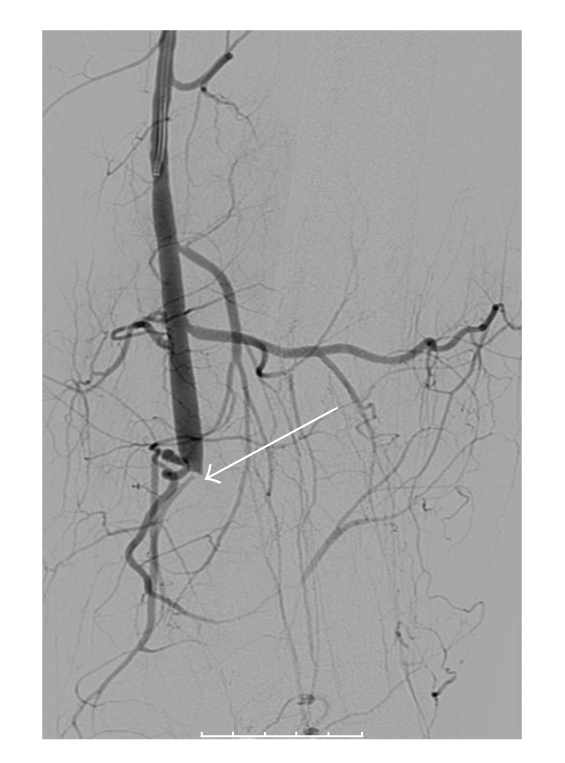
Angiogram of the left leg before thrombolysis, showing complete occlusion of the popliteal artery.

An angiogram revealed a thrombotic occlusion of the proximal half of the popliteal artery and a small embolus in the mid-peroneal artery (Figure 1). Intraarterial thrombolysis (Alteplase) was commenced. A 24-hour followup angiogram showed a reduction in the size of the thrombus; however, there was still a stricture at the midpopliteal artery with an irregularity of the wall. The stricture was accentuated with the foot in dorsiflexion and plantar flexion. At 48 hours, the angiogram demonstrated that the thrombus had largely resolved; however, the stricture was still present. The site of stenosis was angioplastied which resulted in a marked improvement in blood flow.

**Figure 2:**
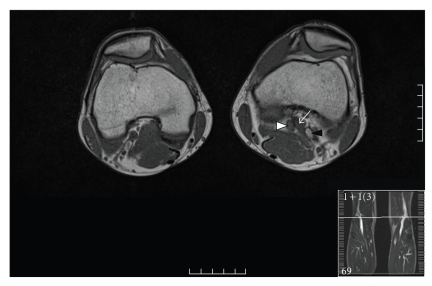
Axial T1-weighted MRI images through both knees at the level of the popliteal fossa showing the medial head of the gastrocnemius muscle (white arrow) going between the popliteal artery (white arrow head) and vein (black arrow head).

An MRI scan was subsequently performed and showed the medial head of the gastrocnemius muscle going between the popliteal artery and vein where it inserted in a more lateral position (Figure 2). Having excluded bilateral disease on the MRI, the patient underwent surgery-division of the medial head of gastrocnemius with autogenous saphenous vein interposition graft. A postoperative duplex scan showed that the vein graft was patent with pulsatile signals. The patient's recovery was uneventful and he was discharged 3 weeks after hospitalisation.

## 3. Discussion

**Figure 3:**
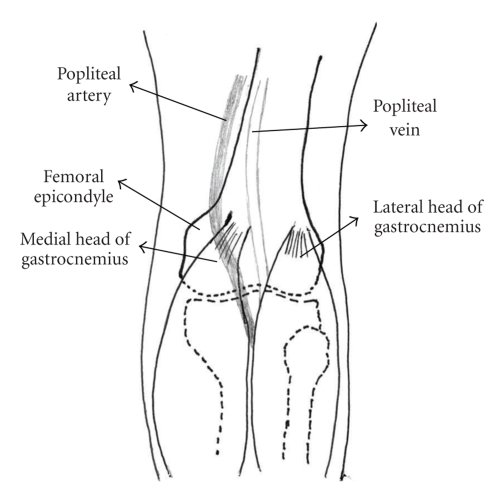
*Type 2 PAES*. The medial head of gastrocnemius muscle is attached more laterally. The popliteal artery courses medially and under the gastrocnemius muscle insertion.

Various classifications of PAES exist in the literature. The most commonly used one was proposed by Whelan [7] and divides this condition into five types (Table 1). Our patient was affected by Type II PAES (Figure 3). 

The presenting symptoms are variable and include leg swelling, aching pain, rest pain, and cramping of the calf. Physical signs are usually absent at rest, until complications develop. In young persons, symptoms are limited to intermittent claudication [4] and this is due to the intermittent compression of the artery during plantar flexion or dorsiflexion. Chronic extrinsic arterial compression leads to vascular microtrauma, early arteriosclerosis, and subsequent thrombosis [10]. Thrombus formation, in the later stages of PAES, may cause complete obstruction of the popliteal artery leading to acute limb-threatening ischaemia; this is commonly seen in patients who have not developed sufficient collateral circulation [11]. Hence, this condition needs to be included in the list of diagnoses in young patients who present with symptoms of peripheral vascular disease.

Angiography is the classical screening and diagnostic tool in PAES [12]. It can demonstrate compression of the popliteal artery with the ankle plantar flexed. Gourgiotis et al. [4] pointed out that an irregularity of the wall of the popliteal artery (in an otherwise normal arterial tree) should also raise suspicion of PAES. Transverse T1-weighted MR imaging is useful to evaluate the aberrant muscular anatomy of the popliteal fossa and also to demonstrate the deviation of the popliteal artery [10]. 

The most common and successful treatment option is muscle division to release the entrapment. If there is significant delay in diagnosis, the popliteal artery may become occluded, stenotic, or aneurysmal [13]. In such complicated cases, division of the anomalous musculotendinous structure and vascular reconstruction is generally required.

## 4. Conclusion

PAES is a rare but potentially limb-threatening condition affecting predominantly young male adults. This syndrome is difficult to diagnose and, therefore, poses a diagnostic pitfall. Awareness of this entity is a prerequisite for correct and prompt diagnosis. Careful history and physical examination is of utmost importance. It should be included in the differential diagnosis of acute popliteal artery occlusion or claudication in young patients with no cardiovascular risk factors. Early surgical intervention to release the popliteal artery is the treatment of choice and is important for good operative outcome.

## Figures and Tables

**Table 1:** Classification of PAES [7–9].

Type 1	PA has an aberrant medial
	course around the MHG which
	has a normal insertion above
	the femoral condyle.

Type 2	MHG is inserted more
	laterally on the distal femur
	but PA not displaced.

Type 3	An aberrant accessory slip of
	MHG slings around and
	surrounds the PA.

Type 4	PA is located deep in the
	popliteal fossa and entrapped
	by the popliteus muscle or
	fibrous bands.

Type 5	Any form of entrapment that
	involves both PA and PV.

MHG: medial head of gastrocnemius, PA: popliteal artery, and PV: popliteal vein.
